# Book Review: Bayesian Statistics the Fun Way: Understanding Statistics and Probability With Star Wars, Lego, and Rubber Ducks

**DOI:** 10.3389/fpsyg.2019.03021

**Published:** 2020-01-15

**Authors:** Jose D. Perezgonzalez

**Affiliations:** School of Aviation, Massey Business School, Massey University, Palmerston North, New Zealand

**Keywords:** bayes, statistics, probability, philosophy, methodology

*Bayesian Statistics the Fun Way* is an engaging introduction to Bayesian inference by Kurt (2019). His main goal of producing “a book on Bayesian statistics that really anyone could pick up and use to gain real intuitions for how to think statistically and solve real problems using statistics” (Carrone, 2019) is certainly achieved. Indeed, the book introduces Bayesian methods in a clear and concise manner, without assuming prior statistical knowledge and, for the most part, eschewing formulations. It explores Bayesian inference in a very intuitive way and with engaging examples—from UFOs to conspiracy theorists, via Lego, crime scenes, Start Wars, email click baits, and funfair rubber ducks—and constrains itself well enough for readers to start applying Bayesian inference from the word go.

The book encompasses three main themes—probability, Bayesian inference, and statistics—plus a couple of small appendixes on R programming and calculus. Among entry-level books, Kurt's is excellent at introducing both Bayesian inference (Ch. 1, 9, 17) and Bayesian statistics (Ch. 5, 8, 15, 16, and 18), less so at introducing probability (Ch. 2–4, 6, and 7—e.g., Phillips, 1973, does it better), at delving into descriptive statistics (Ch. 10–13), and at presenting Bayesian parameter estimation (Ch. 19—which reads as an afterthought, tucked away at book's end).

A big strength of the book is that Kurt restricts analyses and inferences to proportions, mostly relying on the Beta distribution. Although Kurt never explains the reasoning behind such constrain—e.g., Berry does a better job at explaining that “proportions can play a role in essentially any statistical application… [and] the ideas involved in handling this relatively simple kind of data are the same as those for analyzing more complicated measures” (Berry, 1996, p. 167)—the wisdom of such decision allows the reader to engage with the Bayesian framework seamlessly, from prior adjudication to likelihood calculation, all the same touching upon hypothesis testing (Bayes factors), Markov Chain Monte Carlo (MCMC), and credible intervals. Therefore, readers can start applying Bayesian inference straightaway without being swamped by the entire edifice of its potential analytical capability.

Another strength of the book is that Kurt eschews formulations so typical of most Bayesian books—in so doing, it practically contradicts both Kruschke's assertion that “there is no avoiding mathematics when doing data analysis” (Kruschke, 2015, p. 2), and Lambert's moaning that frequentism got the historical upper hand because “many are discouraged from using Bayesian statistics… due to its supposed difficulty and its dependence on mathematics… [while] frequentist statistics sweep their inherent complexity and assumptions under the carpet” (Lambert, 2018, p. 6). This eschewing of unneeded formulations helps make the book straightforward and engaging. And considering that most readers will never use such formulas because a computer will do the pertinent calculations, Kurt has truly instantiated the systemic classification discussed by Pirsig^1991^, p. 173), which, paraphrased, would read as, mathematics and Bayesian inference are not continuous but discrete; although the inferential procedure may be built on the mathematical one, it is not an extension of the latter. Ergo, it is not necessary for a book on Bayesian inference to be clogged with mathematical formulation (or R code, for that matter).

There are a couple of inconsistencies in the book, albeit these are more curious than threatening. For example, Kurt asserts that a hypothesis that predicts the data (well) increases the probability of the data [P(*D| H1, X*) >> P(*D| X*), p. 7], a statement that exemplifies the conjunction fallacy described by Kahneman (e.g., 2011)—I believe Kurt rather wanted to state that the probability of the data will be higher under a good predictive hypothesis than under its absence [P(*D| H1, X*) >> P(*D|* ¬*H1, X*)]. He also ties himself into knots in trying to explain that data inform beliefs, not vice versa, as he uses the likelihood—P(*D | H, X*)—to substantiate that “we change our beliefs according to data… and observations,” and he uses the Bayesian expression—P(*H | D, X*)—to substantiate that “we gather data to support our existing beliefs” (p. 10)—I believe Kurt's explanation will lead the reader to confuse the frequentist P(*D| H*) with the Bayesian P(*H| D*).

All-in-all, I have found only a few truly introductory books capable of initiating naïve readers into Bayesian inference without assuming prior statistical and calculus knowledge. Of those, Phillips's (1973) and Berry's (1996) are out of print, Lambert's (2018) and Kruschke's (2015) are more suitable for readers committed to the Bayesian framework, and Donovan and Mickey's (2019) is formatted as a first-year statistics textbook, which may prove somewhat cringy to a general readership. Kurt's book thus places itself well as a continuation book to McGrayne's *The Theory That Would Not Die* 2011, and as a simple yet complete practical introduction to Bayesian inference and statistics (perhaps even as a stepping stone into other more full-fledged works in the field). Indeed, Kurt's book has been the only reference which gave me full confidence and practical understanding for using Bayesian inference to gain good insights into three problems I had at the time of first reading it and for which I could neither possibly obtain a frequentist answer nor did I get prompted for a plausible resolution by other Bayesian works—albeit I had to supplement such insights with an instantiation of severity philosophy, if only to prevent merely falling into degrees of confirmation (e.g., Mayo, 2018; also Perezgonzalez et al., 2019). In brief, Kurt's *Bayesian Statistics the Fun Way* is a book quite suitable for a crash course in applied Bayesian statistics.

## Author Contributions

The author confirms being the sole contributor of this work and has approved it for publication.

### Conflict of Interest

The author declares that the research was conducted in the absence of any commercial or financial relationships that could be construed as a potential conflict of interest.
